# Research trends in nutrition and exercise for sarcopenic obesity: a bibliometric analysis

**DOI:** 10.3389/fnut.2025.1615101

**Published:** 2025-10-14

**Authors:** Guoqing Zhang, Juncui Hu, Chanjuan Chen, Wenyan Zhu, Yingyi Chen, Yi Cheng, Wen Hu, Zhiyong Rao

**Affiliations:** ^1^Department of Clinical Nutrition, West China Hospital of Sichuan University, Chengdu, China; ^2^Department of Clinical Nutrition, The First People’s Hospital of Yibin, Yibin, China

**Keywords:** sarcopenic obesity, nutrition, exercise, bibliometric analysis, intervention

## Abstract

**Objectives:**

With global population aging and obesity rates rising, the prevalence of sarcopenic obesity (SO) is being rapidly increased, resulting in significant health impacts and making it a growing public health concern in academic circles. Exercise and nutrition are crucial in the etiology, prevention, and management of SO; therefore, bibliometric research in this field can provide key insights for future scientific inquiry.

**Methods:**

The literature concerning on nutrition and exercise in SO published over the past 20 years and indexed in the Web of Science Core Collection database and Scopus database was screened, and bibliometric analyses were subsequently conducted using CiteSpace and VOSviewer.

**Results:**

A total of 658 literatures were selected for bibliometric analysis. A steady rise in publications in this field over 20 years was shown by the results, with major contributions being made by China and the US. The most literatures were published by the University Clermont Auvergne, with Professor Yves Boirie being identified as the most prolific author. The journal *Nutrients* accounted for the most publications. Exercise research appeared earliest, with nutritional intervention gaining attention in recent years. Research frontiers over the past 5 years have primarily focused on three main themes: exercise and nutritional interventions, the exploration of pathophysiological mechanisms, and the establishment of clinical guidelines, with “osteosarcopenic obesity” being a prominent and emerging area of research.

**Conclusion:**

This study provides a comprehensive overview of the current state of nutrition and exercise research in SO.

## 1 Introduction

The concept of sarcopenic obesity (SO), initially introduced by Heber in 1996 (1), has since garnered substantial recognition within the academic community as a salient public health concern, particularly in the context of aging populations (2, 3). This complex pathological condition is characterized by the concurrent and progressive decline in skeletal muscle mass and function, alongside the abnormal accumulation of adipose tissue (2). The escalating global trend of population aging, coupled with the increasing prevalence of obesity, has led to a rapid rise in the incidence of SO. For instance, a study conducted in South Korea among healthy volunteers aged 20 to 80 years reported a SO prevalence ranging from 0.8 to 22.3% in women and 1.3 to 15.4% in men (4). In the United States, the prevalence of SO reaches as high as 28.3% in individuals aged 60 and older, escalating to 66.6% among Mexican Americans (5). A meta-analysis encompassing 86,285 participants revealed the global prevalence of SO in older adults (≥60 years) to be approximately 11%, with a discernible increase observed after the age of 70 (6). Given the rising rates of obesity and sedentary lifestyles among older populations, the prevalence of SO is projected to increase substantially in the coming decade.

SO is closely associated with a spectrum of adverse health outcomes, including metabolic dysfunction, prolonged hospitalizations, geriatric syndromes, diminished quality of life, and elevated mortality risk (7, 8). Despite the expanding body of intervention research dedicated to SO, there are currently no approved pharmacological agents or targeted therapies for its management (9, 10). Consequently, nutritional and exercise interventions have emerged as pivotal strategies for both the prevention and management of SO, and are therefore a significant focus of contemporary research (2, 10, 11).

Bibliometric analysis, a quantitative research method used to evaluate the evolution of a specific field of study, provides a valuable tool for exploring the development of scientific knowledge and its trajectory over time (12). Considering the crucial role of nutrition and exercise in the etiology, progression, prevention, and management of SO, in-depth bibliometric research in this area is expected to provide important insights to inform future research directions. Therefore, this study first employs bibliometric analysis to review the literature on nutrition and exercise in SO research over the past two decades, aiming to summarize the current state of development in this field and provide a basis for exploring future research trends.

## 2 Materials and methods

### 2.1 Literature search

The reporting of this bibliometric analysis conforms to the guidelines outlined in the Preliminary guideline for reporting bibliometric reviews of the biomedical literature (BIBLIO) checklist for biomedical literature (Supplementary Table 1) (13). The Web of Science database and Scopus database were utilized as the primary search platform for this study. We conducted searches in the Web of Science Core Collection (WoSCC) and the complete Scopus database for literature related to nutrition and/or exercise on SO, while aiming to exclude irrelevant publications. The document types were limited to “article” and “review,” the language to English, and the publication period from January 1, 2005, to July 10, 2025. The detailed search strategies are shown in Supplementary Tables 2, 3.

### 2.2 Literature screening

**Figure 1:**
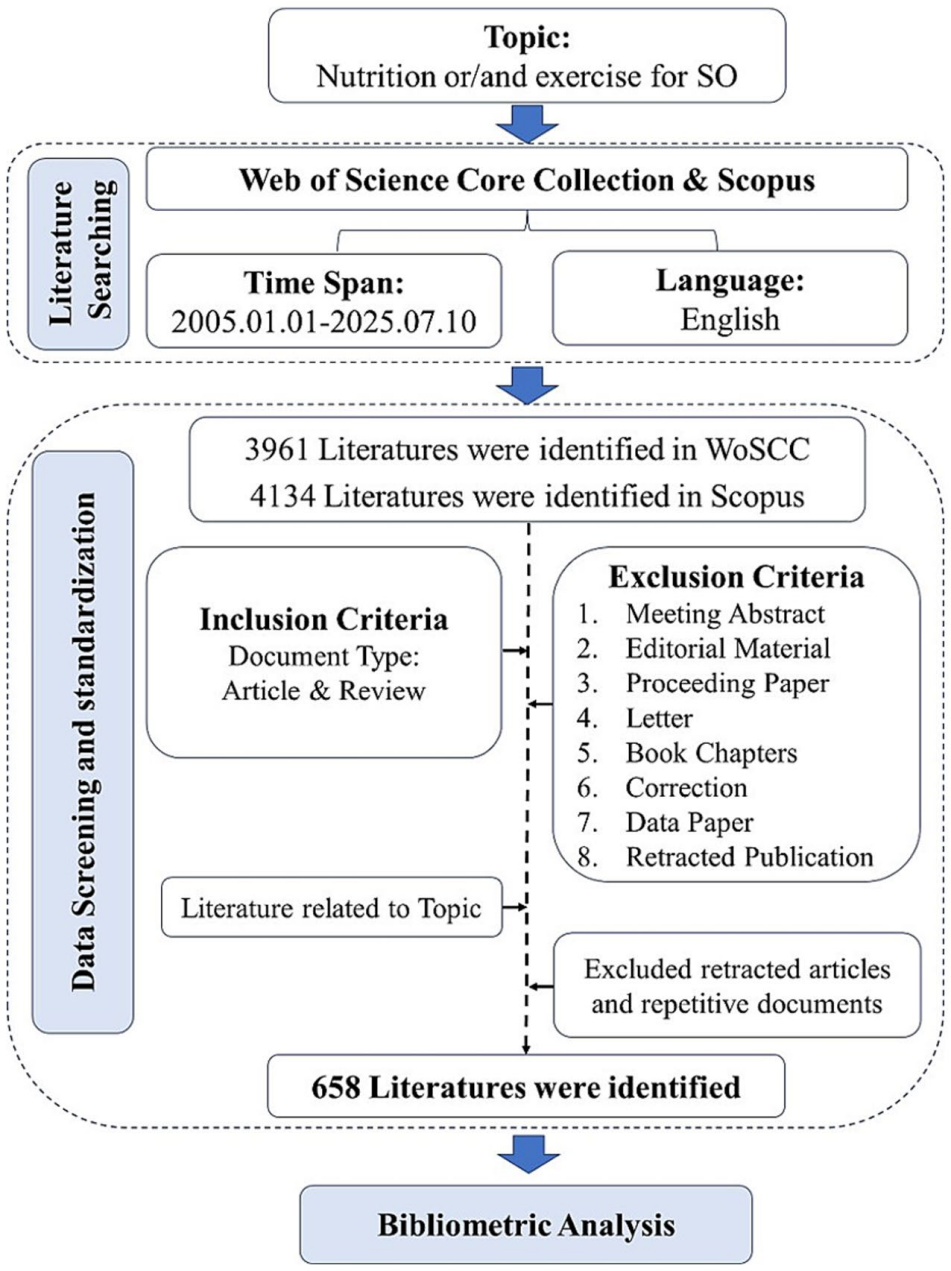
Flow diagram of study selection and data analysis strategies.

The literature screening process is illustrated in Figure 1. The retrieved articles underwent further screening to identify the final set of publications for analysis. Authors (JH and WZ) independently assessed the full text to confirm that the primary focus aligned with our review’s scope, applying the following inclusion and exclusion criteria. Discrepancies were resolved by a third author (GZ). Inclusion criteria: Peer-reviewed articles were included if they substantively examined nutrition and/or exercise in the context of SO. This encompassed a wide range of research, including intervention trials, observational studies, surveys, reviews, and studies focused on etiology, diagnostics, or management. Exclusion criteria: (1) Articles irrelevant to the research topic; (2) Retracted articles and repetitive documents. All records of the selected articles were exported in plain text format.

### 2.3 Data visualization and statistics

Microsoft Excel 2019 was used for analyzing the annual publication trends. VOSviewer and CiteSpace, two scientific mapping tools with complementary strengths in bibliometric visualization, were employed. VOSviewer 1.6.20 and CiteSpace 6.4.R1 were used to perform visual analyses of authors, institutions, countries, journals, and keywords in the included literatures.

## 3 Results

### 3.1 Annual publication trend

**Figure 2:**
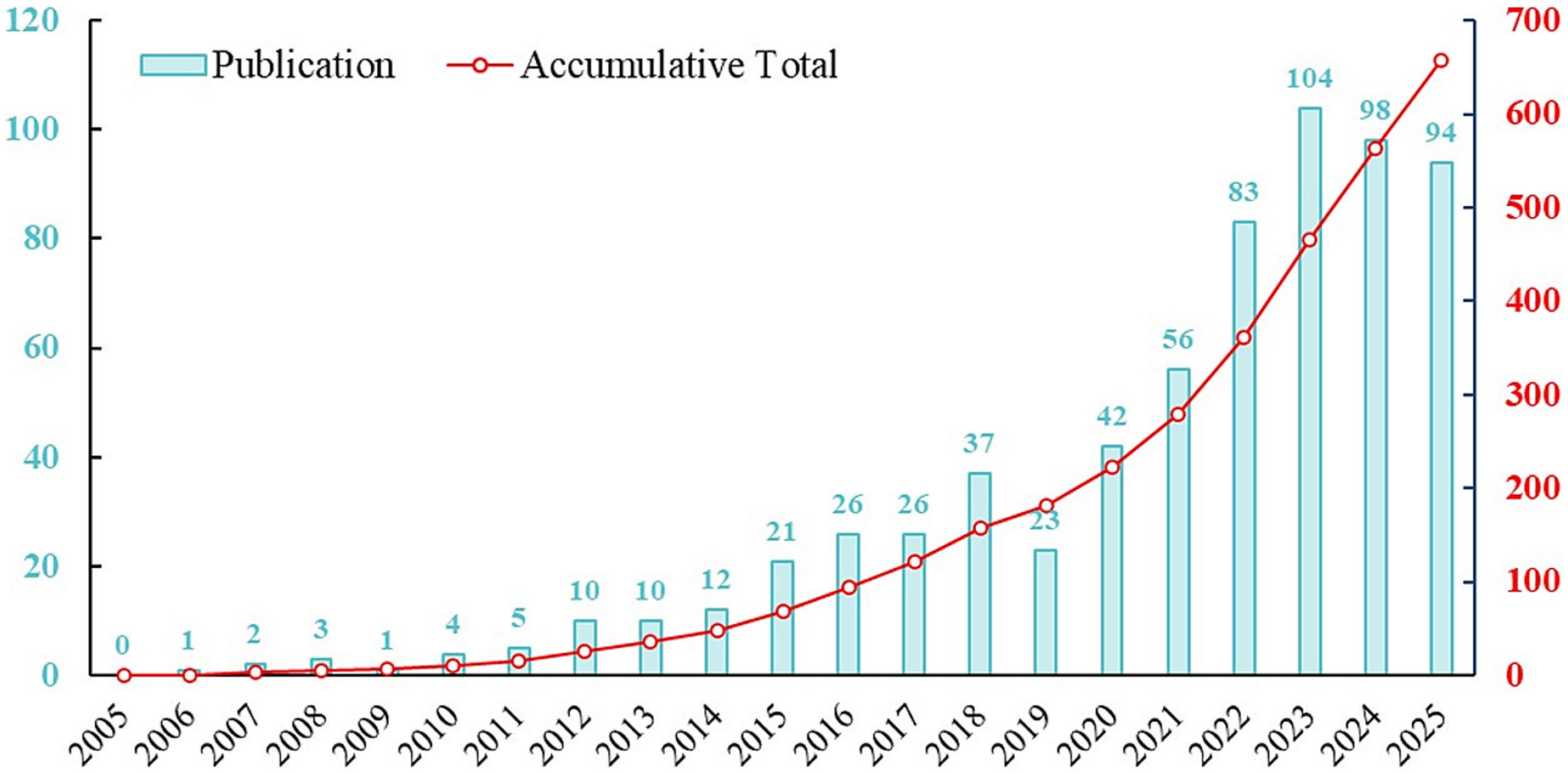
The annual and cumulative number of publications from 2005 to 2025.

Based on the inclusion/exclusion criteria, 658 articles and reviews were ultimately identified from the WoSCC and Scopus (Figure 1). Figure 2 illustrated the annual and cumulative publication trends of studies of SO concerning nutrition and exercise. Prior to 2014, the number of publications in this field was relatively low and exhibited a slow growth trend. However, as clearly visible in the steep rise of the curve in Figure 2, a notable and rapid increase in publication output was observed over the subsequent decade. In 2023, the annual publication counts surpassed 100 for the first time, with 104 documents being recorded. Although a slight decrease in annual publication volume was observed in 2024 compared to 2023, the overall cumulative publication number continued to demonstrate a steady upward trend. Notably, as of July 10, 2025, the number of published literatures (*n* = 94) had already approached the total for the entire year of 2023 (*n* = 98). This substantial upward trend in scholarly productivity highlights the rapid growth and increasing interest in this research area.

### 3.2 Analysis of national publications

**Figure 3:**
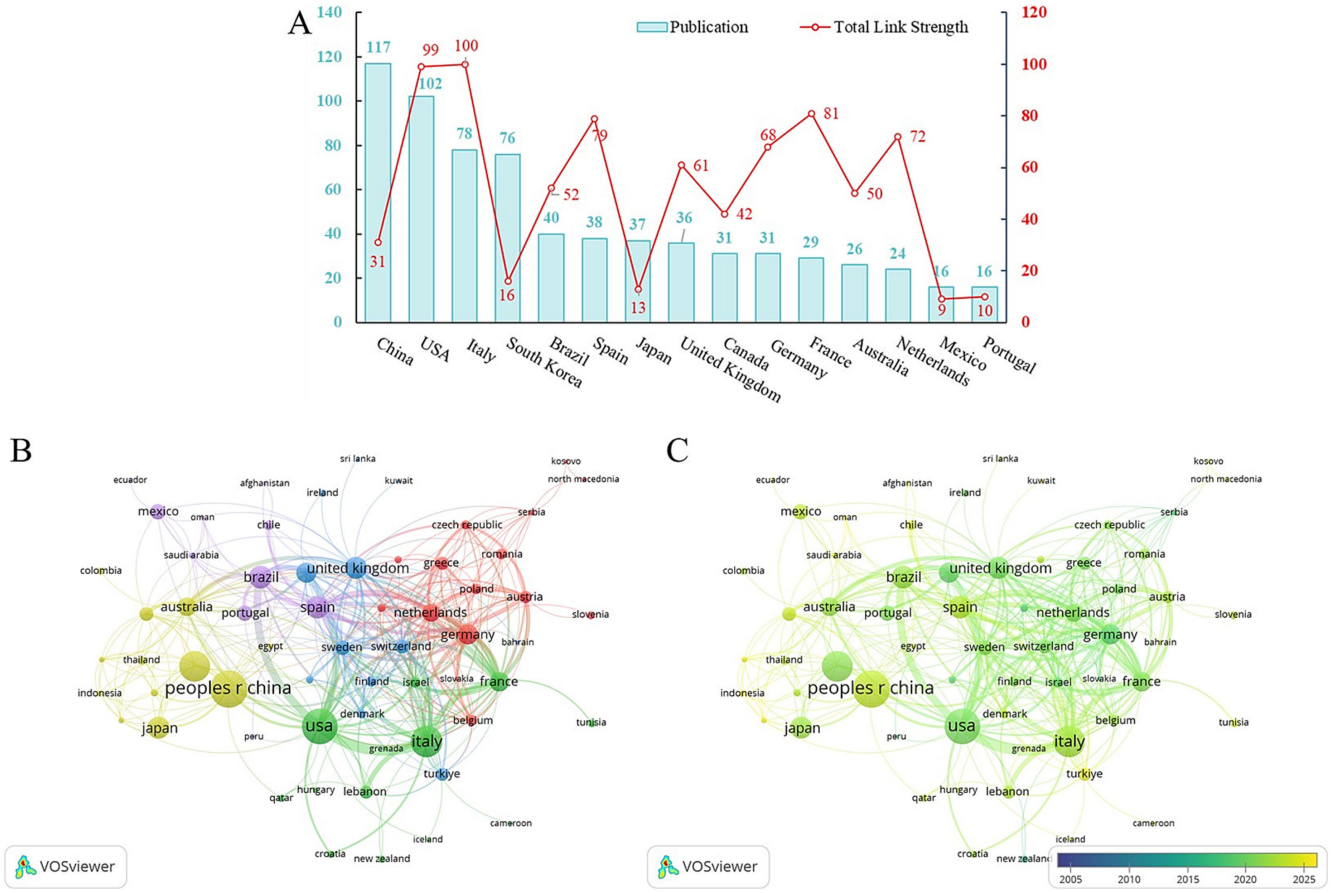
Analysis of countries/regions. **(A)** Top 15 national publications and their average citation frequency. **(B)** Network visualization map of countries/regions collaboration. **(C)** Time-overlapping network visualization map of countries/regions.

An analysis of the publications by country/region was conducted to identify the most contributing countries/regions in this field. It was determined that a total of 67 countries/regions were represented across all publications. As shown in Figure 3A, the top 15 countries/regions ranked by publications, with the China ranking first with 117 publications, followed by United States (102 publications), Italy (78 publications), and South Korea (76 publications). The remaining countries/regions had fewer than 50 publications each. Total link strength is employed to quantify the density and activity of connections for each country within the international cooperation network. It is noteworthy that while China has the highest publication output, its total link strength is only 31. In contrast, Italy and the United States lead the rankings with total link strengths of 100 and 99, respectively. This key finding, clearly visible in Figure 3A, indicates that although China is a major producer of research, it is less central to the international collaboration network compared to other top-ranking countries. Furthermore, Spain, the United Kingdom, Germany, France, and the Netherlands all exhibit a total link strength exceeding 60, indicating their prominent roles in high-intensity international collaboration (Figure 3A).

Furthermore, a visualization analysis of the collaboration network among countries/regions was performed (Figure 3B). In this visualization, the size of the circles represented the publications of each country/region, while the color of the circles indicated the collaboration intensity among different countries/regions within the clustering network. The results revealed that five collaboration clusters were formed among the 67 countries/regions. The largest of these, the red cluster, is comprised of 15 countries and visually highlights a major, tightly-knit international research hub. Additionally, a timeline-based visualization analysis of country/region-specific publications (Figure 3C) demonstrated that in recent years, countries with higher publications in this field were primarily concentrated in Asia and Europe, such as China, Chile, Thailand, Indonesia, Turkey, and Spain. This trend visibly confirms the growing diversification and internationalization of scholarly output in this research area.

### 3.3 Analysis of institution publications

To further explore the contributions of different institutions to studies of SO related to nutrition and exercise, an analysis of publications by institution was conducted. The results revealed that a total of 1,245 institutions worldwide had published relevant research literature in this field. As presented in Table 1, University Clermont Auvergne ranked first with 14 publications. Taipei Medical University and University of Alberta followed closely with 13 publications. Amsterdam University of Applied Sciences, Friedrich Alexander University of Erlangen-Nuremberg and University of Verona were tied for third place, each with 11 publications. However, the top three institutions in terms of total link strength were the University of Trieste, the Université Clermont Auvergne, and the Amsterdam University of Applied Sciences, with scores of 51, 49, and 38, respectively.

**Table 1:** Top 13 institutions for publications.

Rank	Institution	Country	Documents	Total link strength
1	Universite Clermont Auvergne	France	14	49
2	Taipei Medical University	China	13	7
3	University of Alberta	Canada	13	25
4	Amsterdam University of Applied Sciences	Netherlands	11	38
5	Friedrich Alexander University of Erlangen Nuremberg	Germany	11	33
6	University of Verona	Italy	11	20
7	Beirut Arab University	Lebanon	9	8
8	Kyung Hee University	South Korea	9	0
9	Medical University of Graz	Austria	9	30
10	University of Trieste	Italy	9	51
11	Florida State University	USA	8	4
12	Monash University	Australia	8	10
13	Sichuan University	China	8	3

### 3.4 Analysis of journals

The 658 literatures included in this study were disseminated across 282 distinct journals. The top 11 journals, as ranked by publications, are detailed in Table 2 and Supplementary Figure 1. Notably, the three journals with the highest number of published articles were *Nutrients* with 62 literatures, followed by *Clinical Nutrition* (18 literatures) and the *Journal of Cachexia Sarcopenia and Muscle* (16 literatures). It is pertinent to note that all three journals were situated within the first quartile (Q1) of the Journal Citation Reports (JCR), highlighting that a significant portion of this research is published in high-impact, high-quality venues. The journal with the highest impact factor was the *Journal of Cachexia Sarcopenia and Muscle*, with an impact factor of 9.1 in 2024, indicating its leading position and influence within the field.

**Table 2:** Top 11 journals for publications.

Rank	Citing journal	Documents	IF^a^	JCR
1	Nutrients	62	5.0	Q1
2	Clinical Nutrition	18	7.4	Q1
3	Journal of Cachexia Sarcopenia and Muscle	16	9.1	Q1
4	BMC Geriatrics	15	3.8	Q2
5	Frontiers in Nutrition	13	5.1	Q1
6	International Journal of Molecular Sciences	13	4.9	Q1
7	Current Opinion in Clinical Nutrition and Metabolic Care	12	3.5	Q2
8	Experimental Gerontology	12	4.3	Q1
9	Clinical Interventions in Aging	9	3.7	Q2
10	Frontiers in Endocrinology	9	4.6	Q1
11	Scientific Reports	9	3.9	Q1

a IF, impact factor; IF in category according to Journal Citation Reports (2024).

### 3.5 Author impact analysis

A total of 3,353 authors contributed to research pertaining to SO concerning nutrition and exercise. As shown in Table 3 and Supplementary Figure 2, Yves Boirie emerged as the most prolific author, with a total of 11 publications, followed by John A. Batsis (10 publications) and Wolfgang Kemmler (10 publications). Among them, four authors—Fukui, Michiaki, Hamaguchi, Masahide, Okamura, Takuro, and Boirie, Yves—have a total link strength exceeding 50. This metric reflects their central and influential positions within the research network, underscoring the fact that a small number of key researchers are driving a substantial proportion of the high-intensity collaborative efforts in this field.

**Table 3:** Author impact analysis.

Rank	Author	Documents	Total link strength
1	Boirie, Yves	11	50
2	Batsis, John A.	10	33
3	Kemmler, Wolfgang	10	35
4	El Ghoch, Marwan	8	33
5	Itani, Leila	8	33
6	Liao, Chun-De	8	20
7	Weijs, Peter J. M.	8	46
8	Eglseer, Doris	7	33
9	Fukui, Michiaki	7	59
10	Hamaguchi, Masahide	7	59
11	Huang, Shih-Wei	7	20
12	Kohl, Matthias	7	26
13	Kreidieh, Dima	7	31
14	Liou, Tsan-Hon	7	20
15	Okamura, Takuro	7	59
16	Rossi, Andrea P.	7	25

### 3.6 Analysis of keywords

#### 3.6.1 Frequency analysis of keywords

**Figure 4:**
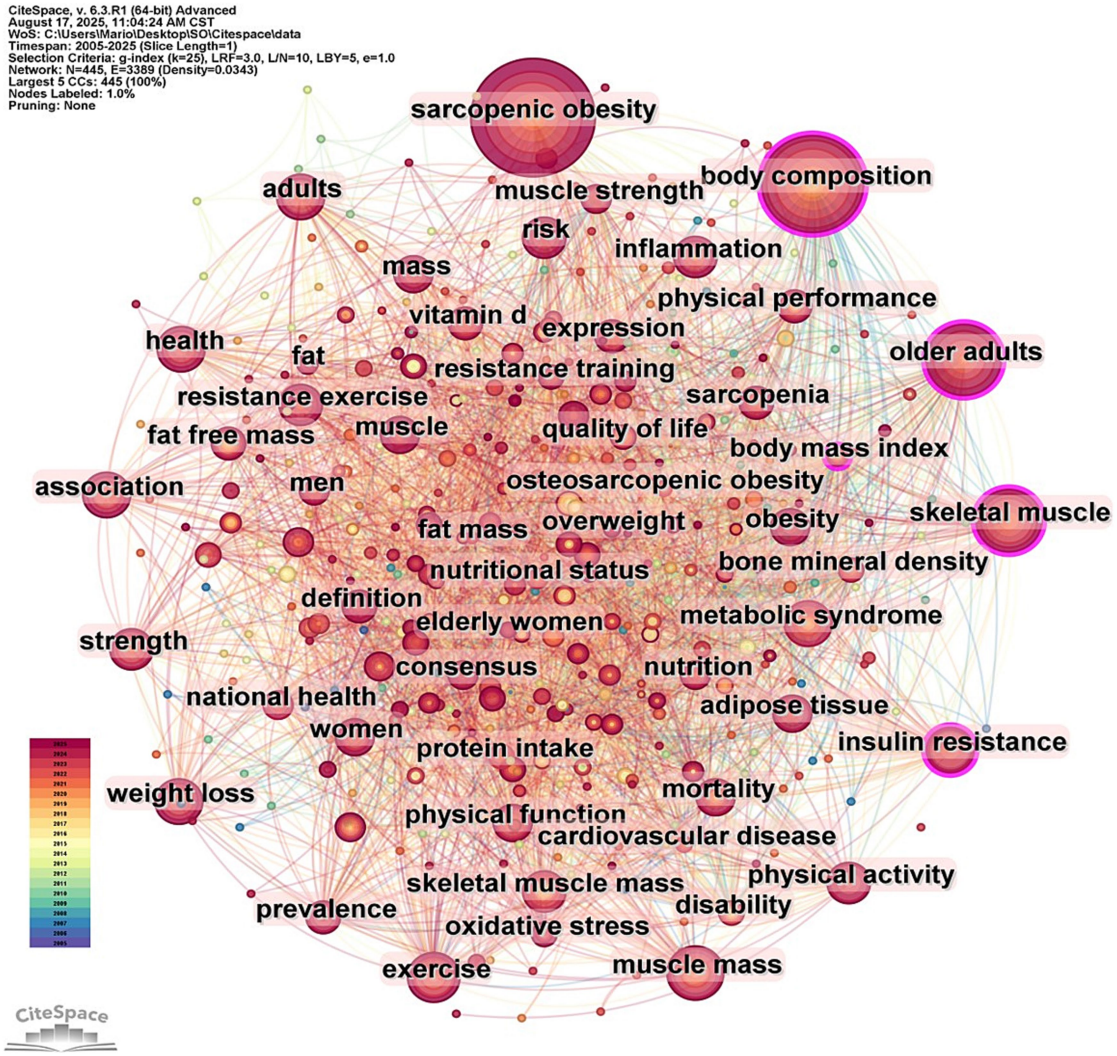
Keyword co-occurrence network.

In the analysis of 658 literatures, a total of 445 keywords were identified. Among these, 20 keywords exhibited a frequency of occurrence exceeding 55 times (Supplementary Table 4). The visualization map of all keywords is presented in Figure 4, wherein node size corresponds to keyword frequency. In the visualization map, 445 nodes and 3,389 edges are observed, indicating the formation of 3,389 co-occurrence relationships among the keywords. As shown in Figure 4 and Supplementary Table 4, four keywords were observed with frequencies surpassing 100 times: “sarcopenic obesity” (321 times), “body composition” (234 times), “older adults” (147 times), and “insulin resistance” (133 times). Among them, “body composition,” “body mass index,” “skeletal muscle” and “insulin resistance” exhibit centrality >0.1 (Supplementary Table 5), underscoring their pivotal roles as structural bridges within the keyword co-occurrence network.

#### 3.6.2 Analysis of keyword clusters

**Figure 5:**
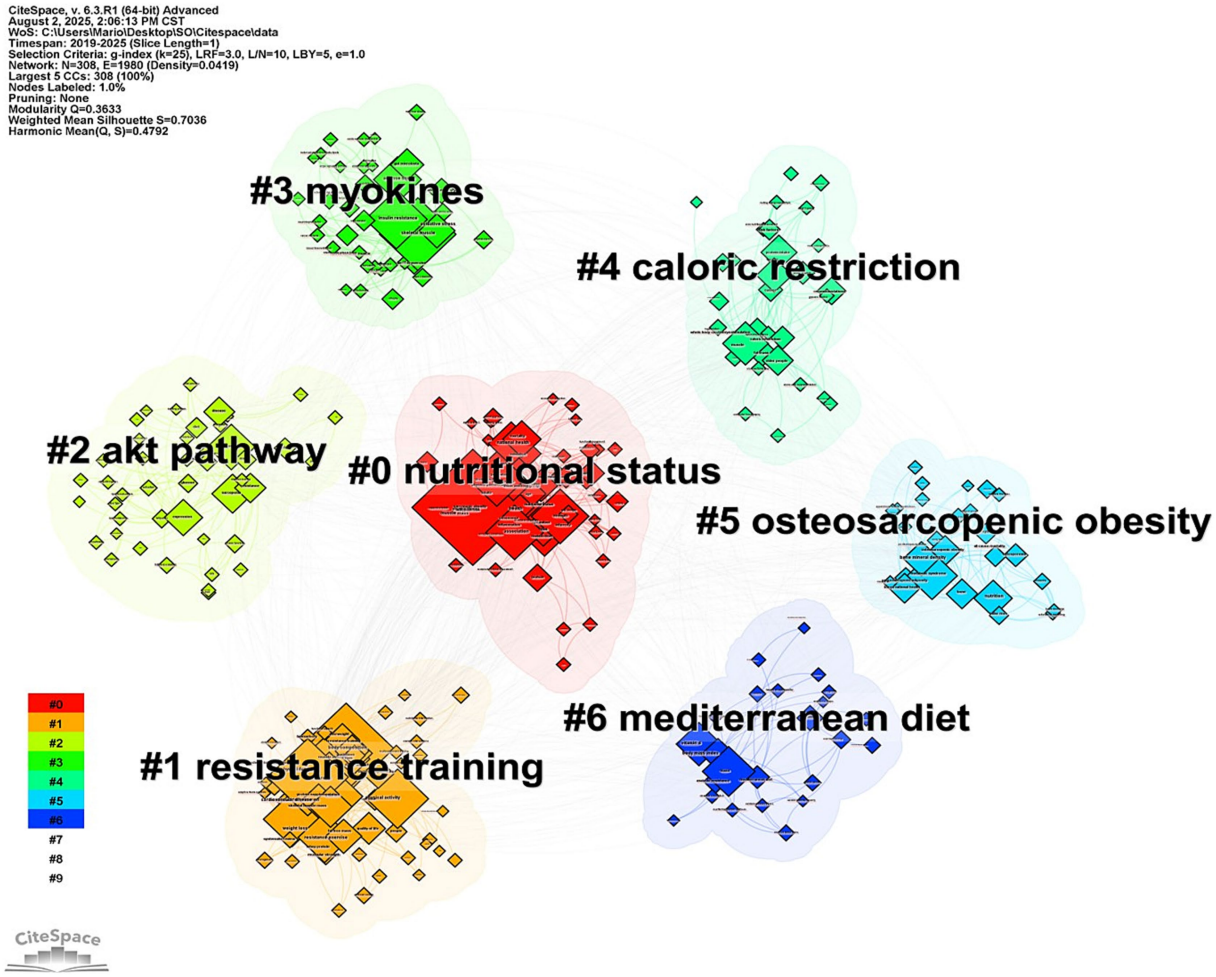
Keyword clusters related to the topic.

To assess the clustering quality, two key metrics were employed: modularity (*Q* value) and mean silhouette coefficient (*S* score). The *Q* value exceeding 0.3 indicates a significant modular structure, whereas the *S* score surpassing 0.7 demonstrates high intra-cluster homogeneity and good separation between clusters. Figure 5 reports a *Q* value of 0.3633 and a *S* score of 0.7036, confirming a robust and well-defined clustering structure, and the results were convincing. Using CiteSpace for keyword clustering, we identified seven main major thematic clusters (Figure 5): “nutritional status,” “resistance training,” “akt pathway,” “myokines,” “caloric restriction,” “osteosarcopenic obesity (OSO)” and “Mediterranean diet.”

#### 3.6.3 Analysis of keyword trends emerging

**Figure 6:**
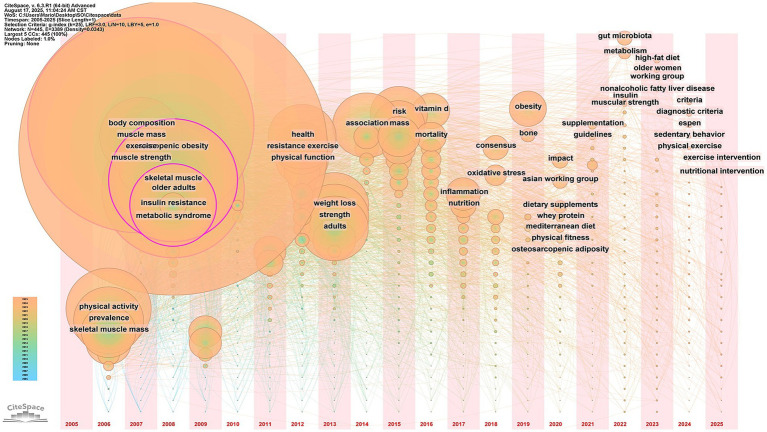
Time-overlapping co-occurrence analysis network of keywords.

To analyze emerging research trends, a timeline visualization of keyword evolution was generated (Figure 6). The visualization indicated that recent research priorities (past 5 years) in this field became increasingly multifaceted and clustered around three main themes: (1) lifestyle and therapeutic interventions for SO, highlighted by new keywords such as “exercise intervention,” “nutritional intervention,” “sedentary behavior,” “physical exercise,” “high-fat diet,” “physical fitness,” “Mediterranean diet,” “whey protein,” “dietary supplements,” and “supplementation”; (2) explorations into pathophysiology and biological mechanisms, with “gut microbiota,” “metabolism,” “nonalcoholic fatty liver disease,” “muscular strength,” and “osteosarcopenic adiposity (OSA)” as representative terms; and (3) the establishment of clinical guidelines and standards, marked by the introduction of key terms like “criteria,” “diagnostic criteria,” “ESPEN,” “working group,” “guidelines,” and “Asian Working Group.”

**Figure 7:**
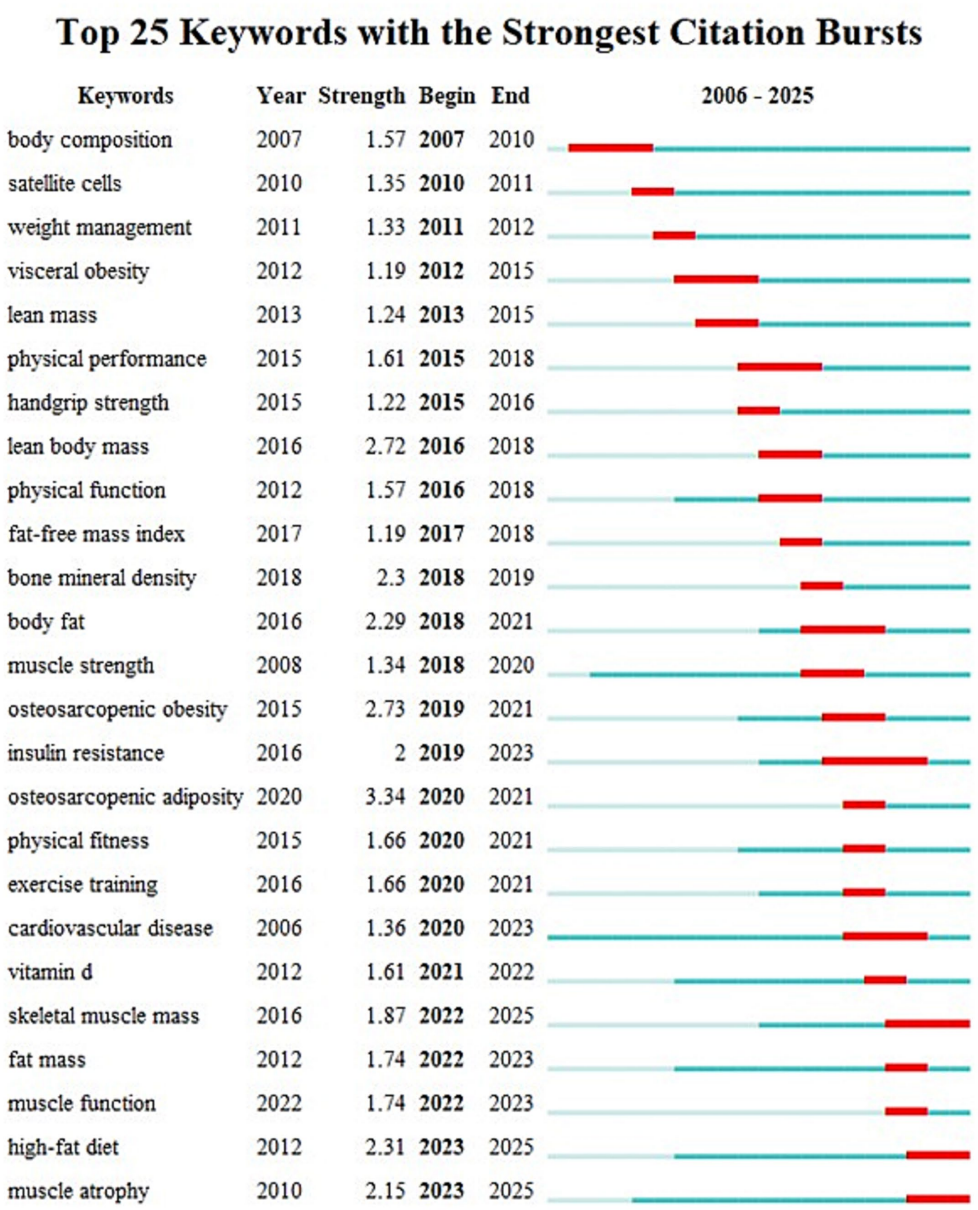
The 25 keywords with the strongest citation bursts.

Keyword burst strength a significant indicator of research frontiers is illustrated in Figure 7 which displays the top 25 keywords by the strongest citation burst with “osteosarcopenic adiposity” and “osteosarcopenic obesity” exhibiting the highest burst strength indicating they are the intensely studied hotspots in this field. Notably the burst duration of “skeletal muscle mass” “high-fat diet” and “muscle atrophy” until 2025 indicates that these are currently the focus of research.

## 4 Discussion

SO, an age-related condition, is projected to experience a further increase in incidence rates, paralleling the global demographic shift towards an aging population (14). Characterized by a complex comorbidity profile, initial research endeavors may have predominantly focused on discrete disease entities, such as obesity or sarcopenia. Consequently, only 658 literatures pertaining to nutrition and exercise in SO were identified over the past two decades. However, a notable surge in research interest concerning nutritional and exercise-based strategies for SO has been observed within the last decade, signifying a period of accelerated advancement in this field. This emerging trend can be attributed to several contributing factors. Firstly, the escalating global prevalence of aging has driven heightened awareness regarding geriatric health imperatives. Secondly, the increasing emphasis on personal well-being is closely associated with improved economic conditions and enhanced living standards. Furthermore, the widespread adoption of non-invasive diagnostic modalities, such as InBody devices (15), has facilitated the convenient identification of SO prevalence and its potential deleterious effects among populations by clinicians and researchers. Accumulating evidence has consistently demonstrated the substantial benefits of nutritional and exercise interventions for individuals with SO (16, 17). As a result, research institutions and investigators worldwide have significantly augmented their support for studies related to nutritional and exercise therapies for SO, thereby propelling the rapid evolution of this domain.

The geographical distribution of countries with the highest publications was predominantly concentrated within Europe, North America, and several countries of Asia. Notably, China and the United States exhibited a dominant publication volume, thereby signifying their pivotal contributions and leading status in this research domain. From a chronological perspective, European and North American nations, including the United States, the United Kingdom, the Netherlands, France and Germany, initiated research in this field at an earlier stage. However, the emergence of Asian countries, exemplified by China, Thailand, and Indonesia, alongside Chile, Turkey, and Tunisia, illustrated the growing trend of diversification and internationalization within SO research. Although China ranks first in publication volume, its total link strength is markedly lower than that of several Western countries, including the United States, Italy, Spain, the United Kingdom, Germany, France, and the Netherlands. This suggests that the latter nations engage in more frequent and intensive transnational collaborations within the international research network. Given this disparity between publication volume and collaboration intensity, especially for emerging research nations, policymakers should focus on fostering strategic international partnerships. This can be achieved by proactively seeking out and formalizing research agreements and joint funding programs with established leaders in the field, such as the United States and European nations. At the same time, institutions should encourage researcher mobility and exchange programs for both students and faculty. This dual approach will create a more dynamic and integrated global research ecosystem, ensuring effective cross-border knowledge sharing.

While both the United States and China demonstrated high publication productivity, the University Clermont Auvergne in France was identified as the institution with the highest publication output. Specifically, Professor Yves Boirie, affiliated with this university, possessed the highest individual publications, thus highlighting his substantial contributions to this field. Professor Yves Boirie specializes in endocrine and metabolic diseases and leads a research team entitled “Diet, muscle health and Sarcopenia.” This team has demonstrated considerable scholarly influence in the domains of metabolic disorders and sarcopenia. Furthermore, Professor John A. Batsishas garnered widespread attention for his long-term research on the synergistic impact of obesity and sarcopenia on health outcomes (9). In parallel, Professor Wolfgang Kemmler concentrated on nutritional and exercise interventions for individuals with sarcopenic obesity (18, 19). The collective work of these two scholars has been pivotal in advancing the field.

Researchers typically select journals that align closely with their research themes and content for manuscript submissions. Statistical analysis reveals that publications in this field are predominantly concentrated in journals dedicated to nutrition, muscle, geriatrics, and metabolism. In terms of publication volume, *Nutrients*, *Clinical Nutrition*, and the *Journal of Cachexia Sarcopenia and Muscle* are the top three. Regarding impact factor, however, the leading positions are occupied by the *Journal of Cachexia Sarcopenia and Muscle*, *Clinical Nutrition*, and *Frontiers in Nutrition*. These findings offer a valuable reference for scholars in related fields when making decisions about journal submission.

Keyword analysis as an instrument for elucidating the dynamic evolution of a research domain effectively reveals the core tenets and developmental trajectories within a specific field. In this study the high-frequency keywords identified primarily pertained to the characteristics of SO and its associated interventions. The high-frequency and high-centrality keywords such as “body composition” “body mass index” “skeletal muscle” “insulin resistance” “metabolic syndrome” and “skeletal muscle mass” signified that the field had evolved beyond simple obesity. Instead it encompassed a complex syndrome in the elderly characterized by the interplay of decreased muscle mass and function abnormal accumulation of adipose tissue and metabolic disorders centered around insulin resistance. This syndrome itself serves as a unifying target for all exercise and nutritional interventions. Keywords such as “physical activity” “exercise” and “resistance exercise” emerged prior to 2012 indicating an early research focus on the impact of physical activity interventions on SO. With the progressive advancement of research nutritional interventions have gained prominence evidenced by the significant increase in the prevalence of the keywords “vitamin D” “high-fat diet” “Mediterranean diet” “whey protein” “dietary supplements” and “supplementation” after 2016. This trend reflects the growing recognition of the value of nutritional management in the context of SO. However it is important to note that a recent analysis of research hotspots shows keywords related to exercise intervention are still experiencing a burst indicating that exercise intervention remains a key area of current research. Furthermore researchers are increasingly combining exercise with nutritional interventions (20, 21) suggesting this comprehensive health model is likely to become a future research hotspot and trend.

The pathophysiology of SO is remarkably complex, fundamentally revolving around a multi-faceted negative crosstalk between muscle and adipose tissue (22). This detrimental interplay involves a spectrum of mechanisms, ranging from classic pathways like chronic low-grade inflammation and oxidative stress to altered Akt signaling and dysregulated inter-organ communication, as evidenced by abnormal myokine secretion. More recently, imbalances in the gut microbiota have emerged as another critical contributor to this condition. Notably, key lifestyle interventions, particularly nutrition and exercise, appear to exert their therapeutic effects by modulating these specific pathophysiological targets. For instance, physical exercise has been shown to ameliorate SO by engaging the IGF-1/Akt/mTORC1 and MAPK/PGC1α signaling pathways (23). Besides, a high-fat diet has been demonstrated to induce SO in naturally aged rats via the gut-trimethylamine N-oxide (TMAO)-muscle axis (24). Conversely, supplementation with milk or taurine has been shown to improve SO symptoms in obese mice by favorably modulating the gut microbiota (3, 25). However, despite this relatively clear framework, several critical questions warrant further investigation. Future research must prioritize elucidating the precise causal relationships and intricate interaction networks among these various contributing factors. Additionally, developing personalized intervention strategies tailored to distinct pathological phenotypes and understanding the long-term synergistic effects of combined interventions at the molecular level remain imperative directions for future exploration.

Notably, the emergence of NAFLD, OSA, and OSO as prominent keywords and independent research clusters signals a significant paradigm shift. This reflects an evolution in academic understanding from the traditional muscle-fat “two-compartment model” to a more complex “three-component model,” which acknowledges the intricate crosstalk between multiple organ systems. For instance, NAFLD shares common pathophysiological drivers with SO, such as dietary habits and physical inactivity (26), and studies have confirmed a significant correlation between them (27). The co-occurrence of NAFLD and SO may elevate the risk of cardiovascular events and all-cause mortality. While a definitive therapeutic approach for this comorbidity remains elusive, lifestyle interventions that include nutritional and exercise modifications are a potentially effective management strategy (26). Besides, a growing body of evidence indicates a frequent co-occurrence of osteoporosis or osteopenia with sarcopenic obesity in recent years. This confluence has led to the clinical concept of OSO (28, 29). To more precisely characterize the body’s fat distribution and deposition beyond the simplistic evaluation of obesity using the Body Mass Index, researchers have proposed a terminological shift from “OSO” to “OSA.” This nomenclatural evolution is crucial for capturing the complex pathophysiology of this syndrome, as it underscores the detrimental impact of visceral and ectopic fat accumulation on metabolic health, the development of comorbidities, and mortality rates (28). The transition from SO to OSO/OSA highlights the systemic and interrelated pathophysiological links among age-related declines in muscle, adipose, and bone tissues. This shift provides a more comprehensive framework for understanding and intervening in the multi-tissue deterioration observed in aging populations (30). Contemporary research in the field of OSO/OSA emphasizes the critical role of nutrition and exercise in both the development and management of this condition. It is understood that physical activity and nutritional interventions can exert synergistic benefits in the prevention and treatment of OSA/OSO by simultaneously modulating bone remodeling, muscle metabolism, and lipid homeostasis (31–33).

Interestingly, newly emerging keywords in recent years have focused on a new theme: clinical guidelines and standards. In 2022, the European Society for Parenteral and Enteral Nutrition (ESPEN) and European Association for the Study of Obesity (EASO) proposed a definition and diagnostic criteria for sarcopenic obesity (34), however, several challenges persist in their practical application. For example, variations in measurement outcomes may arise from the utilization of different body composition assessment tools, such as dual-energy X-ray absorptiometry (DXA) and bioelectrical impedance analysis (BIA), and even more innovative approaches like ultrasound. The applicability of these tools also varies across diverse ethnic groups, age cohorts, and sexes, thereby introducing complexity to the standardization of SO diagnosis. Furthermore, the current diagnostic criteria for SO are primarily predicated on research data derived from Western populations, and their applicability may be limited in individuals with differing ethnicities, body habitus, lifestyles, and cultural backgrounds. A substantial body of evidence-based research is still lacking to support unified diagnostic standards (35). Given the complexity of SO, the absence of precise and well-established diagnostic criteria has resulted in significant heterogeneity in the prevalence data reported globally, owing to the diverse diagnostic standards and assessment tools employed across different studies (7). While the efforts of ESPEN and EASO provide a foundational framework for SO diagnosis, further research and standardization efforts are imperative to ensure diagnostic accuracy and consistency in clinical and research settings. Future investigations should prioritize the evaluation of the applicability of diagnostic criteria across diverse populations and aim to validate and optimize existing standards through large-scale clinical trials.

## 5 Strengths and limitations

This study is to employ a bibliometric approach to systematically review the developmental trajectory and knowledge landscape of exercise and nutrition in the field of SO. It’s important to acknowledge the inherent limitations of bibliometric analysis. Primarily, its descriptive nature allows for the identification of research trends but does not replace the evidence-confirming role of systematic reviews or meta-analyses in evidence-based medicine, which are crucial for assessing the methodological quality of analyzed research. Furthermore, bibliometric studies tend to emphasize publication quantity and collaboration networks, potentially lacking depth in evaluating the quality of clinical evidence. Additional potential biases include the reliance on two major English-language databases, WoSCC and Scopus. This selection may exclude significant publications found in other databases or literature not indexed within these platforms. Moreover, focusing solely on English-language literature might lead to overlooking valuable non-English research or findings from regional databases. Despite these limitations, this study presents several distinct strengths. By leveraging visual clustering techniques, we transform complex literature data into intuitive representations of research hotspots and evolutionary trends. This provides clear macroscopic insights and valuable directional references for future research in this area.

## 6 Conclusion

This bibliometric analysis systematically examined the literature on nutrition and exercise-related themes in SO over the past two decades, identifying key contributing countries, institutions, authors, journals and keywords. The growing focus on these aspects underscores the critical role of nutrition and exercise in the prevention and management of SO. A significant limitation in current research is the lack of consensus on the definition and diagnostic criteria for SO, hindering comparability and standardization. Therefore, establishing unified diagnostic criteria is crucial for advancing research in this area. Furthermore, the study highlights a strong link between SO and various adverse health outcomes, including increased cardiovascular disease risk and reduced quality of life. Consequently, increasing attention is being gained by the importance of nutrition and exercise as effective interventions. Future research should focus on the following aspects: (1) unifying the definition and diagnostic criteria of SO to enhance research comparability; (2) investigating specific exercise modalities and nutritional interventions that are most beneficial for older adults with SO; (3) expanding the scope beyond SO to include OSO/OSA and exploring the interventional effects of exercise and nutrition within these more complex, multi-system contexts.
